# MXene-based functionalized platforms for high-performance MALDI-TOF MS: application in early-stage bloodstream infection biomarker screening

**DOI:** 10.3389/fbioe.2026.1658138

**Published:** 2026-01-23

**Authors:** Wenjia Quan, Yingpo Qiu, Lv Yang, Hao Wang

**Affiliations:** Department of Laboratory Test, Yinzhou No. 2 Hospital, Ningbo, Zhejiang, China

**Keywords:** biomarker screening, bloodstream infection, MALDI-TOF MS, metabolomics analysis, MXene

## Abstract

**Introduction:**

This study developed a MALDI-TOF MS metabolomics analysis method based on MXene nanomaterial functionalization platform for early diagnosis of bloodstream infections (BSI). Currently, BSI detection mainly relies on methods such as blood culture, PCR, and single biomarkers (such as PCT, CRP), which have problems such as long detection time, low sensitivity, and insufficient specificity. Therefore, it is urgent to establish a high-throughput detection technology that is fast, sensitive, and capable of multidimensional analysis.

**Method:**

This study synthesized and characterized MXene nanomaterials, and utilized their ultra-high specific surface area and controllable surface functional groups to construct MXene matrices, significantly improving the enrichment and ionization efficiency of serum metabolites. We used this platform to perform metabolic profiling analysis on 50 BSI positive samples and 50 non BSI control samples, and analyzed the data using principal component analysis (PCA), orthogonal partial least squares discriminant analysis (OPLS-DA), and heatmaps.

**Result:**

The platform achieved an area under the curve (AUC) of 0.981, a sensitivity of 92%, and a specificity of 96% in BSI diagnosis, demonstrating superior performance compared to traditional single biomarkers. Further screening identified multiple potential metabolic markers (M/Z=203.64, 206.75, 218.67, 220.70), all of which had AUCs higher than 0.969.

**Discussion:**

This study not only confirmed the application potential of MXene in mass spectrometry, but also provided a highly sensitive and high-throughput metabonomics technology platform for early screening of infectious diseases. This progress is expected to promote the transformation of BSI diagnosis from single indicator detection to multidimensional metabolic fingerprint analysis.

## Graphical Abstract

**Figure:**
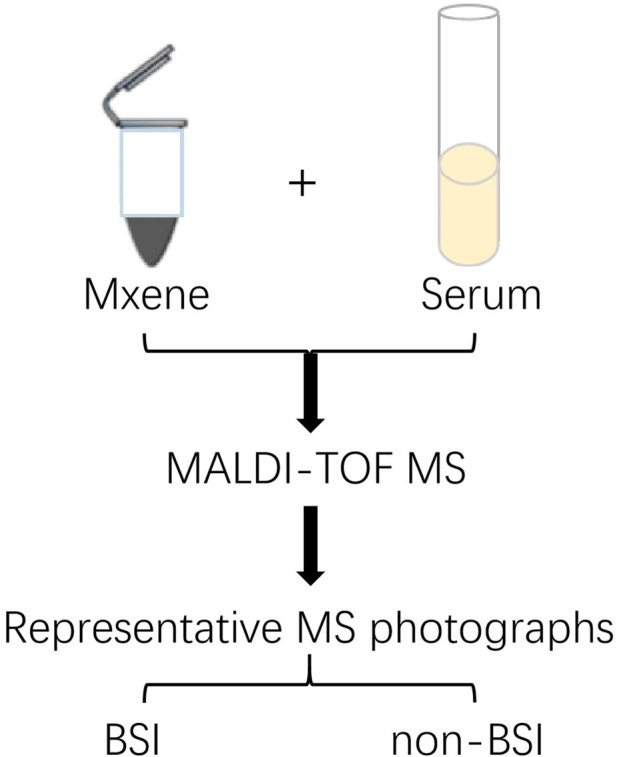

## 1 Introduction

Bloodstream infection (BSI), a serious systemic infectious disease, often leads to sepsis and multiple organ dysfunction syndrome (MODS) (Mervyn et al., 2016; Kristina E et al., 2020; Laura et al., 2021). Despite consuming significant medical resources, the mortality rate for BSI remains high, posing a substantial medical burden. It has become one of the major global public health challenges, making early diagnosis essential for improving patient prognosis and reducing mortality (Mical et al., 2019; Jean-François et al., 2020). However, traditional diagnostic methods such as blood culture, PCR technology, and single biomarker detection have notable limitations: blood culture is time-consuming (typically 24–72 h) and its sensitivity is compromised by antibiotic use. PCR technology, although faster, relies on the genetic information of known pathogens, limiting its effectiveness in complex infection scenarios. Single biomarker-based detection strategies fail to account for the complex molecular network regulation of the disease and are easily influenced by factors such as patient age, underlying conditions, and infection type, leading to significant limitations in sensitivity and specificity (Christophe et al., 2013; Ramon et al., 2017; Fei et al., 2024). For instance, procalcitonin (PCT) levels can be abnormally elevated in noninfectious inflammation, resulting in a false-positive rate of up to 20%–30% (Philipp et al., 2017). The complexity of biomarker interpretation is further highlighted in drug-induced conditions like carfilzomib-induced thrombotic microangiopathy, where markers such as LDH and ADAMTS13 activity must be carefully contextualized (Weilun et al., 2024). Hence, the development of new diagnostic technologies with rapid, high sensitivity, and high specificity has become an urgent clinical need. Recently, matrix-assisted laser desorption/ionization time-of-flight mass spectrometry (MALDI-TOF MS) has shown unique advantages in pathogen identification and metabolomic analysis (Andrew E et al., 2013; Verhoeven et al., 2015). Its high-throughput and high-resolution capabilities enable the simultaneous capture of thousands of metabolite profiles, making it a crucial tool for metabolomics research (Michelle S S et al., 2019). For example, quasi-targeted metabolomics using such platforms has successfully identified predictive metabolites and potential drug candidates in β-thalassemia mouse models (Xianfeng et al., 2024). As a two-dimensional transition metal carbide/nitride material, MXene has garnered significant attention in the field of mass spectrometry due to its ultra-high specific surface area, excellent laser absorption capabilities, and chemical tunability (Shijie et al., 2025; Vivek et al., 2021). The abundant functional groups on the surface of MXene (such as -O, -F) enhance the adsorption and desorption efficiency of analytes and optimize the ionization process through interface engineering, significantly improving detection sensitivity and reproducibility. This principle of material-enhanced detection sensitivity parallels strategies used in nanomedicine, where the precise design of prodrug nanoassemblies has been shown to drastically improve pharmacokinetic profiles and therapeutic outcomes (Chengcheng et al., 2025).

## 2 Experimental

### 2.1 Synthesis and characterization of MXene nanomaterials

Synthesis of MXene Ti_3_C_2_ with reference to and improvement of existing methods (Umay et al., 2022): Slowly add 1 g of Ti_3_AlC_2_ to a polytetrafluoroethylene beaker containing 20 mL of 40% HF and stir the reaction at 35 °C for 24 h. After the reaction is complete, centrifuge the resulting suspension at 3,500 rpm for 5 min, collect the precipitate, and wash it repeatedly with deionized water until the pH of the supernatant is greater than 5.0. The obtained multilayer Ti_3_C_2_T_x_ was vacuum dried at 60 °C for 24 h. Subsequently, add 100 mg of Ti_3_C_2_T_x_ to 10 mL of 1% tetramethylammonium hydroxide (TMAOH) solution and stir for intercalation and fluorohydroxyl exchange. After centrifugation, redispersed the precipitate in 50 mL of deionized water and sonicated for 1 h to achieve sufficient exfoliation. To ensure inter-batch repeatability of the product, repeated centrifugation and ultrasonic dispersion three times. Finally, centrifuge the dispersion at 2000 rpm for 30 min and collect the supernatant to obtain a few-layer Ti_3_C_2_ (f-Ti_3_C_2_) dispersion.

Scanning electron microscopy (SEM, Zeiss Sigma 360) was used to characterize the morphology and structure of MXene. After 5 min of sonication, the MXene suspension was drop-cast onto a conductive substrate and air-dried. The dried samples underwent 45 s of platinum sputtering (GMC-1000) before SEM analysis for morphology and elemental mapping. SEM was typically operated at an accelerating voltage of 5–20 kV, with magnifications ranging from ×20,000 to 500,00x, and the working distance was adjusted (approximately 5–10 mm) to achieve high-resolution images of surface morphology. For EDS elemental mapping, the distribution of elements such as Ti, C, F, and O on the two-dimensional morphology was analyzed.

Fourier-transform infrared spectroscopy (FTIR) was recorded in transmission mode using a Thermo Scientific Nicolet Summit X FTIR spectrometer. A minimal amount of the dried powder sample was thoroughly mixed and ground with dry potassium bromide (KBr) powder in an agate mortar. The mixture was then pressed into a transparent pellet under vacuum. Spectra were collected in the wavenumber range of 400–4,000 cm^-1^ with a spectral resolution of 4 cm^-1^, averaging 32 scans per spectrum to improve the signal-to-noise ratio. A background spectrum of pure KBr was collected and automatically subtracted from the sample spectrum. Raman spectra were acquired using a HORIBA LabRAM HR Evolution spectrometer. The powder sample was placed on a glass slide and gently flattened using another slide to create a smooth surface for analysis. Measurements were performed using a 532 nm laser excitation source with a power of 2 mW at the sample surface. The scattered light was dispersed by a 600 grooves/mm grating. Spectra were collected with an exposure time of 10 s (or 50 s for long-duration tests).

XPS analysis was conducted using a Thermo Scientific K-Alpha XPS spectrometer equipped with a monochromatic Al Kα X-ray source (hv = 1486.6 eV). The powder sample was pressed into a pellet and mounted on a sample holder using conductive tape. After transferring into the instrument’s load-lock chamber, the sample was introduced into the analysis chamber under an ultra-high vacuum better than 2.0 × 10^−7^ mbar. The analysis was performed with a 400 μm X-ray spot size, a 12 kV operating voltage, and a 6 mA filament current. Survey scans were recorded with a pass energy of 150 eV and a step size of 1 eV. High-resolution regional scans were obtained with a pass energy of 50 eV and a step size of 0.1 eV. The binding energy scale was calibrated by referencing the C 1s peak (adventitious carbon) to 284.8 eV.

For nitrogen physisorption (BET surface area) detection, the specific surface area was determined from nitrogen adsorption-desorption isotherms measured at 77 K using a Micromeritics ASAP 2460 volumetric adsorption analyzer. Prior to measurement, the sample was degassed under dynamic vacuum at 120 °C for 8 h using the instrument’s dedicated degas station. The Brunauer–Emmett–Teller (BET) method was applied to the adsorption data within the relative pressure (P/P_0_) range of 0.05–0.30 to calculate the specific surface area.

### 2.2 Clinical sample collection

Fifty positive samples of bloodstream infection (BSI) and fifty control samples (non-BSI) were collected. Three milliliters of venous blood were drawn into coagulant tubes, centrifuged (3,000 × g, 10 min) to isolate serum, which was subsequently used for HE4, PCT, and MALDI-TOF MS analysis. Additionally, 2 mL of venous blood were collected into EDTA tubes for immediate CRP measurement and NLR determination. All residual serum specimens were collected post-routine testing and irreversibly anonymized. Written informed consent was waived by the Ethics Committee of Yinzhou No. 2 Hospital (Approval No. 2024KY1610) in accordance with Article 23 of China’s Regulations on Ethical Review of Biomedical Research (2016) and CIOMS/WHO Guideline §4.3.

### 2.3 MALDI-TOF MS detection

Mass spectrometry analysis was conducted using MALDI-TOF MS (MetaDx). To prepare the samples, 50 μL of serum was mixed with 150 μL of ethanol, thoroughly vortexed, and centrifuged at 15,000 × g for 10 min. Subsequently, 1 μL of the serum supernatant was deposited dropwise onto the steel plate, allowed to air dry, and overlaid with 1 μL of MXene substrate solution. The MXene substrate solution (3 mg/mL) was prepared by dispersing MXene in deionized water. The acquisition parameters were set to positive ion mode with a laser frequency of 60 Hz, accumulating 1000 shots with a maximum of 100 acquisitions per point. Data processing was performed using the Default Low Mass method set.

### 2.4 Single biomarker detection

The HE4 test was conducted using a fully automated chemiluminescence immunoassay analyzer (Abbott i-2000) along with the corresponding reagents. The PCT test utilized a chemiluminescence immunoassay analyzer (Anthem A6200) and the associated reagents. The CRP test was carried out on a fully automated protein-specific analyzer (Shenzhen Pumon PA990) with its respective reagents. The NLR test was performed on a hematology analyzer (UniCel DxH 800) using the appropriate reagents.

### 2.5 Data analysis

Principal component analysis (PCA), orthogonal partial least squares discriminant analysis (OPLS-DA), volcano plot, and heatmap analysis were plotted using MetaboAnalyst 5.0 (https://www.metaboanalyst.ca/home.xhtml). Each MZ feature with a fold change >1.5 and a t-test threshold p-value <0.05 was in the volcano plots. SPSS was used to plot ROC curves and calculate AUC values.

## 3 Results

**FIGURE 1:**
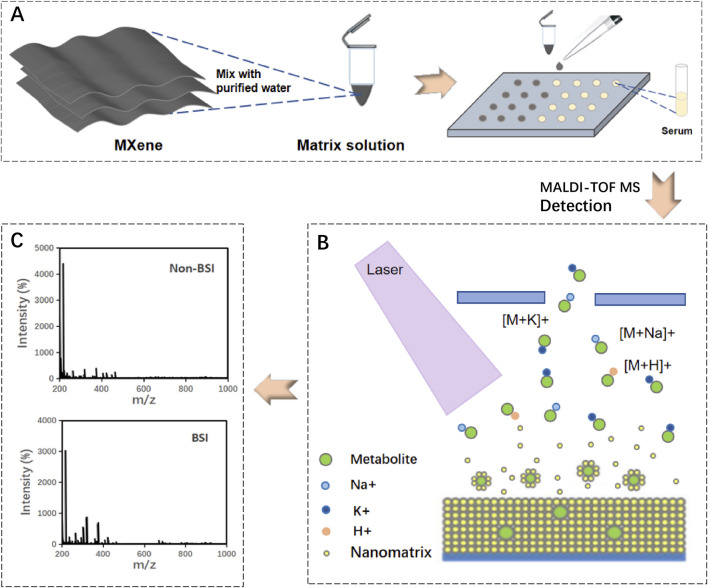
Schematic illustration of MXene-based MALDI-TOF MS detection. **(A)** Handling of matrix materials. **(B)** MALDI-TOF MS analysis of metabolites in clinical samples. **(C)** Representative mass spectrometry (MS) photographs.

In this study, the MXene matrix was utilized with MALDI-TOF MS technology to screen for bloodstream infections. As depicted in Figure 1A, the MXene solution was applied to air-dried serum on a steel plate and subsequently analyzed using MALDI-TOF MS. The mechanism of MALDI-TOF MS is illustrated in Figure 1B. Various metabolites in serum samples are dispersed within the matrix material. Upon laser irradiation, the matrix absorbs the laser energy, facilitating the ionization of metabolite molecules. These charged ions are then detected, and the metabolic fingerprint of the sample is determined based on the mass-to-charge ratio of the ions. A typical spectrum is shown in Figure 1C. Through comprehensive and in-depth analysis of metabolic fingerprints, effective diagnosis of blood infections was achieved.

**FIGURE 2:**
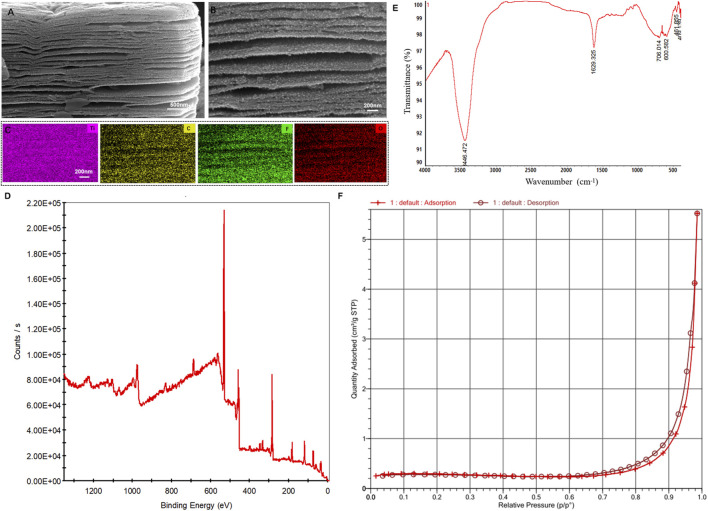
Characterizations of MXene. **(A,B)** Scanning electron microscopy (SEM) image. **(C)** Elemental mapping analysis of MXene, including Ti, C, F, and O. **(D)** The XPS spectrum of MXene. **(E)** The FTIR spectrum of MXene. **(F)** The Nitrogen Physisorption (BET Surface Area) detection of MXene.

Scanning electron microscopy (SEM) characterization revealed the morphology and structure of MXene nanomaterials (Figures 2A,B), which exhibited distinct two-dimensional layered structures. Additionally, EDS elemental mapping displayed the element distribution within MXene, indicating a uniform distribution of Ti, C, F, and O (Figure 2C; Supplementary Figure S1). The unique two-dimensional layered structure and surface chemical properties of MXene enhance the adsorption and desorption efficiency of metabolites. XPS spectrum (Figure 2D), FTIR spectrum (Figure 2E) and the Nitrogen Physisorption (BET Surface Area) detection (Figure 2F) of MXene also was tested.

**FIGURE 3:**
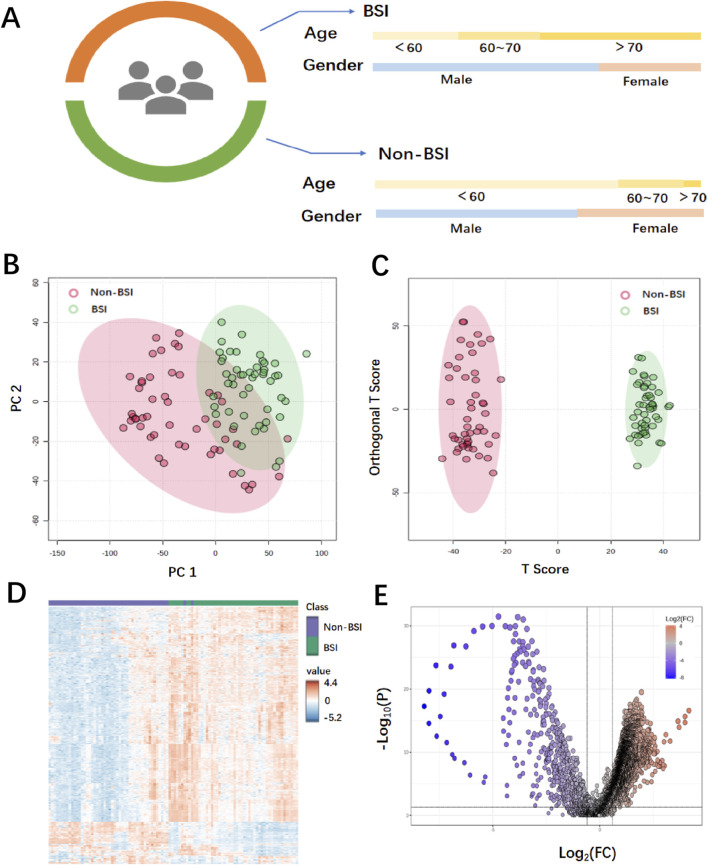
Metabolic analysis of BSI and Non-BSI samples on the MALDI-TOF MS platform. **(A)** Clinical information of serum samples. **(B)** Principal component analysis (PCA). **(C)** Orthogonal partial least squares discriminant analysis (OPLS-DA) analysis. **(D)** Heatmap analysis. **(E)** Volcano plot.

To validate the clinical performance of the MXene nanomatrix applied to the MALDI-TOF MS platform for screening bloodstream infections, we tested 50 positive samples of bloodstream infection (BSI) and 50 samples from a control group (Non-BSI). The sample information is shown in Figure 3A and Supplementary Table S1. To distinguish BSI from Non-BSI, we conducted classical unsupervised principal component analysis (PCA) and supervised orthogonal partial least squares discriminant analysis (OPLS-DA). The PCA analysis, shown in Figure 3B, indicates a partial overlap between Non-BSI and BSI, suggesting that PCA cannot fully extract sufficient differential information to distinguish between disease and non-disease states in such complex data. However, using the OPLS-DA method, the data were directionally processed and analyzed. The OPLS-DA analysis, shown in Figure 3C, effectively distinguished all samples, revealing significant differences in metabolites between the groups. OPLS-DA successfully achieved the effective identification of BSI and Non-BSI. A heatmap can intuitively show the differences in metabolite expression patterns. The heatmap analysis of clinical samples, shown in Figure 3D, reveals clear metabolic differences in composition and intensity between the two groups. The horizontal axis of the volcano plot represents the fold change in metabolite concentration between the two groups (log2 FC), while the vertical axis represents the significance of metabolite differences (−log10 p-value). This plot visualizes statistical significance and fold change, aiding in the rapid screening of metabolites with significant differences between the groups (Gary J et al., 2012; Jasmine et al., 2018). The volcano plot shown in Figure 3E displays the upregulated and downregulated MZ panels, with these highly significant metabolites and low p-values serving as potential biomarkers.

**FIGURE 4:**
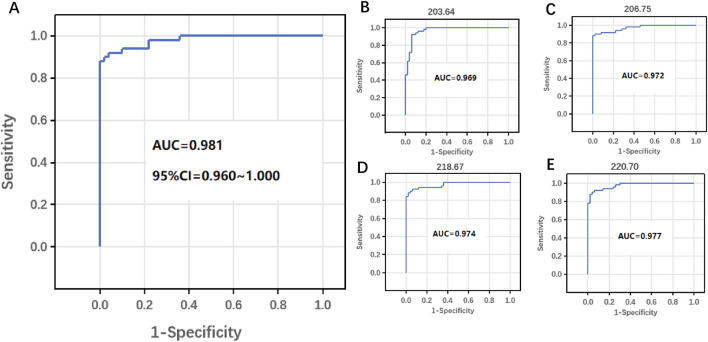
Area under curve (AUC) for MALDI-TOF MS detection. **(A)** AUC for discriminating BSI from Non-BSI. **(B–E)** AUC of MZ = 203.64, MZ = 206.75, MZ = 218.67 and MZ = 220.70.

The serum metabolic fingerprint successfully distinguished between BSI and Non-BSI samples, and the area under the curve (AUC) was further used to evaluate diagnostic performance. As shown in Figure 4A, the AUC of the MALDI-TOF MS platform is 0.981, indicating its superior diagnostic ability in bloodstream infections. Additionally, BSI and Non-BSI clinical groups exhibited significant metabolic differences in MZ values. The MZ values shown in Figures 4B–E are 203.64, 206.75, 218.67, and 220.70, respectively, with corresponding AUC values of 0.969, 0.972, 0.974, and 0.977. These findings suggest that these metabolites may be associated with the occurrence or progression of diseases, and these MZ values can serve as potential biomarker combinations for screening bloodstream infections.

**FIGURE 5:**
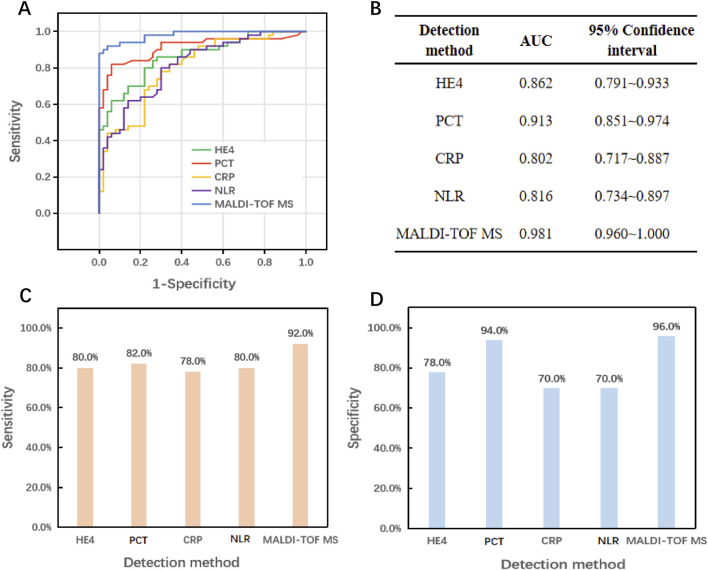
Comparison of the diagnostic efficacy of the MALDI-TOF MS platform with a single marker in differentiating BSI and Non-BSI. **(A,B)** AUC of HE4, PCT, CRP, NLR and MALDI-TOF MS detection. **(C)** Sensitivity comparison. **(D)** Specificity comparison.

Further evaluate the diagnostic performance of the MALDI-TOF MS platform by comparing its detection effectiveness with that of individual biomarkers. Previous studies have demonstrated that human epididymis protein 4 (HE4), procalcitonin (PCT), C-reactive protein (CRP), and the neutrophil-to-lymphocyte ratio (NLR) are valuable in diagnosing bloodstream infections (Vincent et al., 2021; Schuetz et al., 2009; Póvoa et al., 2005). Figures 5A,B present the AUC values for HE4, PCT, CRP, NLR, and the MALDI-TOF MS platform for clinical samples. The AUC values for HE4, PCT, CRP, and NLR were 0.862, 0.913, 0.802, and 0.816, respectively, all of which were lower than the 0.981 achieved by the MALDI-TOF MS platform. Figures 5C,D illustrate the differences in sensitivity and specificity among the various detection methods. The MALDI-TOF MS platform exhibited a diagnostic sensitivity of 92% and a specificity of 96%, outperforming the best single biomarker, PCT, which had a sensitivity of 82% and a specificity of 94%.

## 4 Discussion

Early diagnosis of bloodstream infections (BSIs) remains a significant challenge in clinical practice. Conventional blood cultures are time-consuming (24–72 h) and susceptible to prior antibiotic exposure. Although PCR reduces turnaround time, it requires prior knowledge of pathogen genetic information and struggles with complex infections (Shymma et al., 2025). Single-biomarker strategies (e.g., PCT, CRP) often lack sensitivity and specificity because they overlook the disease’s complex molecular network regulation, which is influenced by patient age, comorbidities, and infection type (Divya et al., 2025; Yosuke et al., 2018; Won-Ho et al., 2017). Therefore, there is an urgent need to develop new diagnostic technologies that are rapid, highly sensitive, and specific.

In recent years, research on nanomaterials for medical testing and drug delivery has advanced substantially. In this study, we successfully developed a functionalized MALDI-TOF MS platform based on MXene nanomaterials and applied it to early metabolomics screening of BSIs. By leveraging MXene’s unique two-dimensional layered structure, ultrahigh specific surface area, and abundant, tunable surface functional groups, the platform enables efficient enrichment and soft ionization of low-abundance serum metabolites, significantly improving analytical sensitivity and reproducibility. Experimental results show a diagnostic sensitivity of 92%, specificity of 96%, and an AUC of 0.981 for BSI, clearly outperforming traditional single-biomarker methods. Comprehensive characterization by XPS, FT-IR, and BET elucidated MXene’s surface chemistry and structural features.

This study demonstrates the clinical utility and diagnostic performance of the MXene-functionalized MALDI-TOF MS platform for early BSI screening. Our platform exploits MXene’s unique physicochemical properties—particularly its high surface area for metabolite enrichment and efficient laser energy absorption/transfer—in synergy with the high throughput of MALDI-TOF MS to achieve rapid, label-free metabolic fingerprinting. Compared with conventional MALDI matrices (e.g., CHCA, SA), the MXene nano-matrix provides a cleaner background and higher ion yields for small metabolites (Fan et al., 2023). Additionally, relative to more complex LC-MS/MS workflows that require lengthy separations and specialized expertise, our platform offers a simplified and rapid workflow (<30 min per sample), suitable for potential point-of-care translation. Future comparative studies will quantitatively benchmark sensitivity, reproducibility, and coverage against gold-standard LC-MS/MS and other emerging nanosheet-assisted MS platforms to fully establish its application in clinical metabolomics testing (Hui et al., 2022; Sen et al., 2023).

Various nanomaterial-assisted LDI-MS platforms have shown promise for disease metabolite analysis in recent years. Although advanced LDI-MS platforms based on diverse nanostructured matrices have been reported—such as graphene oxide derivatives for structural and photochemical studies (Seung-Woo et al., 2022), iron particle–assisted systems for metabolic fingerprinting of diabetic retinopathy (Yu et al., 2023), and ZrMOF/Au hybrids for high-throughput urinary metabolomics (Junyu et al., 2024)—comparative analyses with these recent methods further highlight the advantages of our MXene-based MALDI-TOF MS platform. Our platform differs in several key respects. First, unlike graphene oxide systems that primarily focus on material characterization and photothermal properties, our MXene-functionalized platform is purpose-built for clinical metabolite screening in complex biological samples, delivering an AUC of 0.981 with high sensitivity (92%) and specificity (96%) for BSI diagnosis. Second, relative to iron particle–assisted LDI-MS, our MXene matrix provides enhanced laser absorption and surface functionalization capacity, enabling superior metabolite enrichment and ionization efficiency. Third, while MOF-based hybrids exhibit excellent salt tolerance and reproducibility, our MXene platform offers a unique combination of ultrahigh specific surface area, tunable surface chemistry, and mechanical stability, facilitating reproducible and sensitive detection of low-abundance metabolites without complex sample pretreatment.

Recently, nanomaterial-assisted LDI-MS platforms have demonstrated significant potential in disease metabolite analysis. For instance, graphene oxide and its derivatives have been employed to study photochemical properties, iron particle-assisted platforms have shown clinical utility in metabolic fingerprinting of diabetic retinopathy, and ZrMOF/Au hybrid materials have enabled high-throughput urinary metabolic subtyping of thyroid nodules. The MXene-based platform proposed in this study differs from graphene oxide systems, which primarily focus on material characterization and photothermal properties. Our MXene-functionalized platform is specifically designed for clinical metabolite screening in complex biological samples, achieving an AUC of 0.981 with high sensitivity (92%) and specificity (96%) for bloodstream infection diagnosis.

Compared to iron particle-assisted LDI-MS, our MXene matrix offers enhanced laser absorption and surface functionalization, resulting in superior metabolite enrichment and ionization efficiency. Although MOF-based hybrids exhibit excellent salt tolerance and reproducibility, the MXene platform described here provides a unique combination of ultrahigh specific surface area, tunable surface chemistry, and mechanical stability. This enables reproducible and sensitive detection of low-abundance metabolites without complex sample pretreatment. MXenes possess higher laser energy absorption and photothermal conversion efficiency, which improves ionization efficiency and reduces fragmentation interferences. Their surface functional groups can be chemically modified to target the enrichment of specific metabolites. Additionally, MXenes maintain a high specific surface area while offering better mechanical stability and reusability potential, making them more suitable for high-throughput screening of clinical samples.

## 5 Conclusion

In conclusion, this study not only verified the potential application of MXene in mass spectrometry but also provided an efficient and high-throughput metabolomic analysis platform for the early diagnosis of infectious diseases. The further development of this platform is expected to facilitate the transition of bloodstream infection diagnostics from reliance on single indicators to multidimensional metabolic fingerprint analysis, thereby offering a new technological pathway for the accurate diagnosis and treatment of infectious diseases.

## Data Availability

The datasets presented in this study can be found in online repositories. The names of the repository/repositories and accession number(s) can be found in the article/Supplementary Material.
