# Supplementary material for: MXene-based functionalized platforms for high-performance MALDI-TOF MS: application in early-stage bloodstream infection biomarker screening
Source: Front Bioeng Biotechnol. 2026 Jan 23;14:1658138. doi: 10.3389/fbioe.2026.1658138 (PMC12876243; doi:10.3389/fbioe.2026.1658138)
Supplement: Supplementary file 1 [file Supplementaryfile1.docx]

***Supplementary Information***

**MXene-Based Functionalized Platforms for High-Performance MALDI-TOF MS: Application in Early-Stage Bloodstream Infection Biomarker Screening**


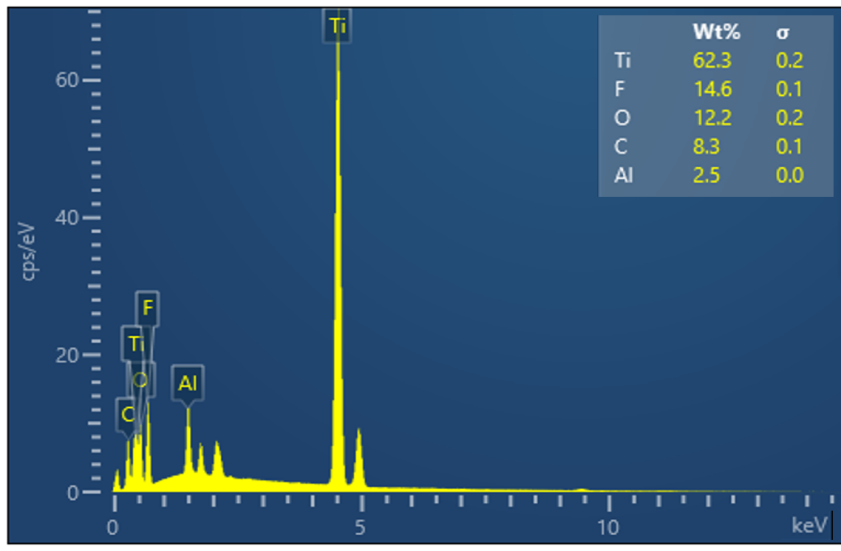


**Supplementary Figure S1.**EDS elemental distribution map of MXene.

**Supplementary Table 1.**Clinical sample information.

| Serial Number | Group | Gender | Age | Serial Number | Group | Gender | Age |
| --- | --- | --- | --- | --- | --- | --- | --- |
| 1 | Non-BSI | Male | 82 | 51 | BSI | Male | 59 |
| 2 | Non-BSI | Male | 21 | 52 | BSI | Male | 72 |
| 3 | Non-BSI | Male | 62 | 53 | BSI | Male | 61 |
| 4 | Non-BSI | Male | 70 | 54 | BSI | Female | 74 |
| 5 | Non-BSI | Male | 45 | 55 | BSI | Male | 62 |
| 6 | Non-BSI | Male | 61 | 56 | BSI | Female | 51 |
| 7 | Non-BSI | Female | 69 | 57 | BSI | Female | 58 |
| 8 | Non-BSI | Female | 43 | 58 | BSI | Male | 61 |
| 9 | Non-BSI | Male | 56 | 59 | BSI | Male | 42 |
| 10 | Non-BSI | Male | 74 | 60 | BSI | Female | 68 |
| 11 | Non-BSI | Female | 34 | 61 | BSI | Male | 76 |
| 12 | Non-BSI | Female | 70 | 62 | BSI | Female | 90 |
| 13 | Non-BSI | Male | 43 | 63 | BSI | Male | 84 |
| 14 | Non-BSI | Male | 37 | 64 | BSI | Female | 38 |
| 15 | Non-BSI | Male | 23 | 65 | BSI | Male | 73 |
| 16 | Non-BSI | Male | 69 | 66 | BSI | Male | 62 |
| 17 | Non-BSI | Female | 45 | 67 | BSI | Male | 70 |
| 18 | Non-BSI | Male | 69 | 68 | BSI | Female | 84 |
| 19 | Non-BSI | Female | 22 | 69 | BSI | Female | 78 |
| 20 | Non-BSI | Male | 19 | 70 | BSI | Male | 72 |
| 21 | Non-BSI | Female | 44 | 71 | BSI | Male | 64 |
| 22 | Non-BSI | Male | 59 | 72 | BSI | Female | 67 |
| 23 | Non-BSI | Male | 32 | 73 | BSI | Female | 93 |
| 24 | Non-BSI | Female | 47 | 74 | BSI | Female | 47 |
| 25 | Non-BSI | Male | 44 | 75 | BSI | Female | 81 |
| 26 | Non-BSI | Male | 48 | 76 | BSI | Male | 70 |
| 27 | Non-BSI | Male | 38 | 77 | BSI | Male | 73 |
| 28 | Non-BSI | Male | 48 | 78 | BSI | Male | 65 |
| 29 | Non-BSI | Male | 22 | 79 | BSI | Male | 87 |
| 30 | Non-BSI | Female | 50 | 80 | BSI | Male | 87 |
| 31 | Non-BSI | Female | 62 | 81 | BSI | Female | 86 |
| 32 | Non-BSI | Male | 53 | 82 | BSI | Female | 84 |
| 33 | Non-BSI | Male | 62 | 83 | BSI | Male | 61 |
| 34 | Non-BSI | Female | 35 | 84 | BSI | Male | 63 |
| 35 | Non-BSI | Female | 56 | 85 | BSI | Female | 86 |
| 36 | Non-BSI | Male | 69 | 86 | BSI | Male | 42 |
| 37 | Non-BSI | Male | 23 | 87 | BSI | Female | 91 |
| 38 | Non-BSI | Male | 72 | 88 | BSI | Female | 27 |
| 39 | Non-BSI | Female | 37 | 89 | BSI | Male | 68 |
| 40 | Non-BSI | Male | 23 | 90 | BSI | Male | 83 |
| 41 | Non-BSI | Male | 58 | 91 | BSI | Female | 38 |
| 42 | Non-BSI | Male | 26 | 92 | BSI | Female | 80 |
| 43 | Non-BSI | Female | 33 | 93 | BSI | Female | 68 |
| 44 | Non-BSI | Female | 29 | 94 | BSI | Female | 78 |
| 45 | Non-BSI | Female | 54 | 95 | BSI | Female | 74 |
| 46 | Non-BSI | Female | 57 | 96 | BSI | Male | 86 |
| 47 | Non-BSI | Female | 37 | 97 | BSI | Male | 70 |
| 48 | Non-BSI | Female | 48 | 98 | BSI | Male | 60 |
| 49 | Non-BSI | Female | 54 | 99 | BSI | Female | 62 |
| 50 | Non-BSI | Male | 63 | 100 | BSI | Male | 77 |
